# The Use of CGH Arrays for Identifying Copy Number Variations in Children with Autism Spectrum Disorder

**DOI:** 10.3390/brainsci14030273

**Published:** 2024-03-13

**Authors:** Agata Kucińska, Wanda Hawuła, Lena Rutkowska, Urszula Wysocka, Łukasz Kępczyński, Małgorzata Piotrowicz, Tatiana Chilarska, Nina Wieczorek-Cichecka, Katarzyna Połatyńska, Łukasz Przysło, Agnieszka Gach

**Affiliations:** 1 Department of Genetics, Polish Mother’s Memorial Hospital-Research Institute, 93-338 Lodz, Poland; wanda.hawula@iczmp.edu.pl (W.H.); lena.rutkowska@iczmp.edu.pl (L.R.); urszula.wysocka@iczmp.edu.pl (U.W.); lukasz.kepczynski@iczmp.edu.pl (Ł.K.); malgorzata.piotrowicz@iczmp.edu.pl (M.P.); tatiana.chilarska@iczmp.edu.pl (T.C.); nina.wieczorek-cichecka@iczmp.edu.pl (N.W.-C.); agnieszka.gach@iczmp.edu.pl (A.G.); 2 Department of Developmental Neurology and Epileptology, Polish Mother’s Memorial Hospital-Research Institute, 93-338 Lodz, Poland; katarzyna.polatynska@iczmp.edu.pl (K.P.); lukasz.przyslo@iczmp.edu.pl (Ł.P.)

**Keywords:** autism spectrum disorders, neurogenetics, copy number variation

## Abstract

Autism spectrum disorders (ASDs) encompass a broad group of neurodevelopmental disorders with varied clinical symptoms, all being characterized by deficits in social communication and repetitive behavior. Although the etiology of ASD is heterogeneous, with many genes involved, a crucial role is believed to be played by copy number variants (CNVs). The present study examines the role of copy number variation in the development of isolated ASD, or ASD with additional clinical features, among a group of 180 patients ranging in age from two years and four months to 17 years and nine months. Samples were taken and subjected to array-based comparative genomic hybridization (aCGH), the gold standard in detecting gains or losses in the genome, using a 4 × 180 CytoSure Autism Research Array, with a resolution of around 75 kb. The results indicated the presence of nine pathogenic and six likely pathogenic imbalances, and 20 variants of uncertain significance (VUSs) among the group. Relevant variants were more prevalent in patients with ASD and additional clinical features. Twelve of the detected variants, four of which were probably pathogenic, would not have been identified using the routine 8 × 60 k microarray. These results confirm the value of microarrays in ASD diagnostics and highlight the need for dedicated tools.

## 1. Introduction

One of the most common neurodevelopmental conditions is autism spectrum disorder (ASD). It is characterized by impairment in social interactions and communication, repetitive stereotyped behavior and restricted interest or activities [1]. The prevalence of ASD is estimated to be about 1 in 100 children worldwide and its more frequent in males [2]. ASD itself is a complex disorder with broad clinical symptoms and severity [3]. Although the symptoms persist to adulthood, the first typical manifestations can be observed in the early years of life, and a diagnosis can usually be made between the second and fourth years of age [4]. Patients with ASD commonly display several additional clinical features. Many present with psychiatric conditions, with the most common being depression, anxiety disorders, attention deficit hyperactivity disorder (ADHD), sleep and eating disorders, and self-injurious behavior [5]. Among children with ASD, the prevalence of intellectual disability (ID) ranges from 30% [6,7] to 70% [8,9]. Approximately 19% of patients with ASD present with epilepsy, and around 60% have EEG epileptiform activity [10]. As in the case of ID, a higher percentage of epilepsy has been noted among patients with autism (11% to as much as 39%) compared to those with ASD [11]. Importantly, about 40% of children with both ASD and ID also have epilepsy [10]. Such a wide diversity of clinical symptoms and the presence of comorbidities may hinder a diagnosis of ASD. It is believed that 70–80% of ASD patients present with isolated ASD, also described as pure or essential ASD, with the remainder demonstrating complex ASD, i.e., accompanied by other comorbidities such as epilepsy, intellectual disability, congenital anomalies, dysmorphia, developmental delay [12,13,14,15].

Although the precise pathogenesis of ASD is not known, it is believed to be heterogeneous, comprising a range of environmental, genetic, and epigenetic factors [3]. Of these, the genetic factors are considered to contribute 25–35% of the final condition [16]. Indeed, to date, hundreds of ASD-related genes have been identified. These genes are associated with various systems which are known to play key roles in autism, such as synapse signal transduction, axon guidance processes and neuronal development, and code for proteins involved in synaptic cell adhesion. Approximately 10% of cases of autism are associated with a genetic syndrome; these include both monogenic disorders such as fragile X syndrome (FXS), Rett syndrome, neurofibromatosis, tuberous sclerosis, and Timothy syndrome, as well as chromosome aberrations such as Angelman syndrome, Down’s syndrome or Cornelia de Lange syndrome [15,17]. The most common monogenetic cause of inherited intellectual disability, learning disability, and ASD is an CGG-expansion type, and the full mutation (>200 CGG repeats) of the *FMR1* gene [18]. FXS occurs in about 2–5% of people with autism, and possibly more than that in ASD. Also, premutation (55 to 200 CGG repeats) in this gene may contribute to the ASD phenotype [19]. The *FMR1* gene encodes for a protein called fragile X messenger ribonucleoprotein, or FMRP, which is crucial in regulating the translation of proteins involved in synaptic plasticity; as such, a deficiency of this protein mostly leads to intellectual deficits and is more prevalent in men [20]. A study of the incidence of CNVs in patients with premutation in *FMR1* by Lozano et al. showed a significantly higher percentage of CNVs (50%) in patients with premutation and neurological disorders. However, the share of CNV in patients with ASD, both with and without additional neurological symptoms, was at a similar level to that noted in patients without any neurodevelopmental disorders, i.e., 11.1%, 15.8%, and 15%, respectively [21]. Interestingly, while epilepsy has a similar prevalence in patients with idiopathic ASD and syndromic ASD, individuals with FXS and seizures were more likely to have ASD than those with FXS but without seizures [22].

In the human genome, one of the most common forms of polymorphism is copy number variation (CNV). In general, a CNV is defined as the deletion or duplication of a section larger than 50 base pairs as a result of structural rearrangement [23]. CNVs can be classified as either benign polymorphisms responsible for population diversity, or pathogenic variants that may cause disease or increase the phenotypic diversity of a condition. Although the vast majority of studies have focused on the influence of single-nucleotide variants (SNVs) in the pathogenesis of various disorders, some have highlighted the role played by CNVs [24,25]. It is estimated that CNVs are responsible for approximately 10% of ASD cases [26].

In both research and clinical diagnostic settings, the standard method of detecting CNVs involves the use of a comparative genome hybridization array (aCGH), the first-tier genetic method for diagnosing inter alia dysmorphia, intellectual disability, delayed milestone, and ASD [27]. While conventional karyotyping typically identifies around 3% of cases, a chromosomal microarray is estimated to detect aberrations in 10 to 20% of patients [28]. The tool is also rapid, accurate, and relatively cost-efficient. A range of array platforms are currently in use, being characterized by different degrees of genome coverage, resolution, and methodology, and the numbers and densities of their probes can influence many of the variations detected by aCGH [24]. The present study uses a dedicated microarray to examine the range of genetic variation among a group of participants with ASD. The aim is to assess the relationship between clinical features and the occurrence of pathogenic and uncertain CNVs in a group of patients with ASD, with or without comorbidities.

## 2. Materials and Methods

The studied cohort included 180 pediatric patients with ASD with an age range of 2.4 to 17.9 years: 138 male and 42 female patients with a median age of six years and eight months at the time of aCGH analysis. These patients were divided into two cohorts. The first group comprised 105 patients with a diagnosis of isolated ASD (80 male, 25 female). The second group comprised 75 patients (58 male, 17 female) with one or more additional congenital anomalies or other clinical features, such as dysmorphia, intellectual disability, or epilepsy. Overall, the male–female ratio in the group was 3.3:1, which is consistent with the higher susceptibility to ASD noted among males in the general population [29].

Of the patients participating in the study, detailed clinical data were available for 94 of the 105 patients with isolated ASD and for 63 of the 75 patients diagnosed with ASD with additional clinical features.

Motor development delay was reported in 40% of cases with isolated ASD and in 50.7% of patients with ASD and additional clinical features.

Among patients with isolated ASD, 68.6% demonstrated delayed speech development or regress of speech, while 11.4% presented a complete lack of speech development. Among those with additional clinical features, 66.6% had delayed or regressed speech development while 4% had lack of speech development.

In addition, behavioral and movement disorders were reported throughout the tested group. Motor hyperactivity, stereotypic movements or dyspraxia were noted in 39% of patients with isolated ASD and in 36% of those with ASD and additional clinical features. Aggressive and self-injurious behavior occurred in 6.7% of patients with isolated ASD, and in 9.3% of those with ASD and additional clinical features.

One of the important additional features accompanying ASD was intellectual disability (ID). Unfortunately, it was not possible to obtain data regarding ID in 21.3% of patients, either due to early age or communication problems. Among the remainder, ID was found in 52%. For 25 of these patients, it was possible to determine the degree of disability: eight had mild ID, 15 moderate ID, and two severe ID.

Epilepsy was noted in 17 of the 75 ASD patients with additional clinical features (22.7%). Additionally, 30.7% of children in this group were found to have dysmorphic features: characteristics such as excessive body weight, decreased muscle tone, and visual and hearing defects were observed to a small extent throughout the group, and macrosomia was also noted.

The patients had been referred from the Genetics Clinic and the Neurology Department of the Polish Mother’s Memorial Hospital Research Institute in the years 2018–2021, and the entire study was performed in the Department of Genetics at the Institute. All parents or guardians of the probands gave their informed consent to participate prior to the genetic procedures. The study was also approved by the Bioethical Committee of the Polish Mother’s Memorial Hospital Research Institute.

Identification of ASD was based on clinical interview, psychiatric, and clinical psychologist consultation and assessment of patients according standardized diagnostic instruments. One of these used in the presented study is the Diagnostic and Statistical Manual of Mental Disorders, Fifth Edition (DSM-5) and the Diagnostic and Statistical Manual of Mental Disorders, 4th Edition (DSM-IV). The patients were assigned a disease code based on the ICD-10 classification. Clinical data available at the time of recruitment were included in the analysis. The patients were referred to our center with a diagnosis made at external specialized psychological and pedagogical counselling centers, which in the Polish health care system deal with clinical diagnosis and support of patients with suspected ASD [30].

DNA was extracted automatically from peripheral venous blood in EDTA using a MagCore^®^ Genomic DNA Whole Blood Kit (TK Biotech, Warsaw, Poland). The concentration and purity of the DNA were assessed by NanoDrop 2000 (Thermo Scientific™, Waltham, MA, USA). The DNA samples were then described and registered in the internal database. The occurrence and extent of CNVs was examined using the whole-genome ASD-dedicated microarray CytoSure 4 × 180 k (Oxford Gene Technology, Oxford, UK) according to the manufacturer’s protocol. The microarray slides were scanned using a SureScan Dx Microarray Scanner (Agilent Technologies, Santa Clara, CA, USA) and the image files were processed with Agilent Feature Extracted 12.1.0.3 software.

The results were analyzed with CytoSure Interpret Software, v.4.10, with the human genomic sequence GRCh37 (hg19) used as a reference. The results were interpreted and classified using the following databases: DGV (Database of Genome Variants), DECIPHER (Database of genomic Variation and Phenotype in Humans using Ensembl Resources), PubMed, OMIM (Online Mendelian Inheritance in Man), ISCA (ClinGen Dosage Sensitivity Curation Page), and ClinVar-NCBI. In the case of VUSs, pathogenic and likely pathogenic variants, the next step was to confirm the variants and establish their inheritance. If family members were available, they were contacted by a clinician for comparative diagnostics. In such cases, the parents of the probands gave their informed consent to take part, and in the case of underage siblings, informed consent was obtained from the guardians.

The comparative diagnostics was performed in one of two ways, depending on the type of CNV: either by aCGH or by fluorescence in situ hybridization (FISH). The latter procedure involves the use of probes targeted at a specific position in the chromosome, and can be used on both interphase cells and metaphase chromosomes. Various chromosome rearrangements, such as deletions, duplications, and translocations, can be detected by hybridizing complementary probes to DNA. Compared to aCGH, the FISH method allows balanced translocations to be identified in the parents, which is important for genetic counseling and estimating the risk for the next pregnancy [31,32]. Briefly, the samples used for FISH were collected in heparin and used for cell culture. The lymphocytes from the cell sediments were then subjected FISH diagnostics according to the manufacturer’s protocol depending on the used probes.

## 3. Results

The variants were assessed based on literature data, public databases such as DGV, DECIPHER, ClinVar, OMIM, and our own internal database. Detected imbalances were classified into five categories according to the ACMG guidelines, viz. pathogenic, likely pathogenic, VUSs, likely benign, and benign [33]. Variants assessed as benign were not reported in the results; only pathogenic and likely pathogenic variants were considered as clinically relevant.

The results indicated the presence of nine pathogenic and six likely pathogenic variants in 14 of the 180 patients, i.e., a prevalence of 7.8%. The frequency of pathogenic and likely pathogenic variants was higher in males. Additionally, 20 VUSs were detected in 16 individuals (8.9%) and five likely benign variants in five patients (2.8%). In total, the reported CNVs included 20 deletions and 20 duplications. The CNV size varied between 284 b and 4.04 Mb. The highest value recorded in the study, two imbalances, was reported for five individuals. One patient had two pathogenic variants, another had one likely pathogenic and one VUS, and three patients had two VUSs.

**Figure 1:**
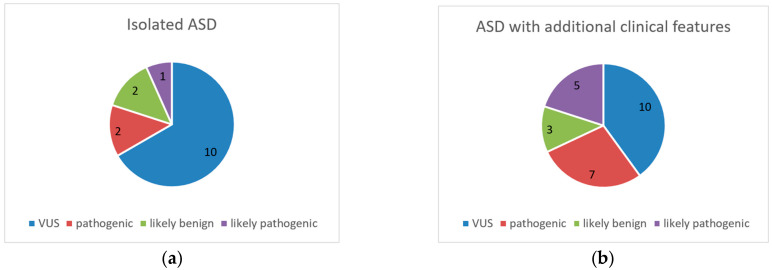
The number of variants identified in each pathogenicity category in patients with isolated ASD (**a**) and ASD with additional clinical features (**b**).

In the group of 105 patients with isolated ASD, two pathogenic variants, one likely pathogenic, 10 VUSs, and one likely benign variant were identified. Among patients with ASD with additional clinical features, six harbored seven pathogenic variants and five individuals had rare imbalances classified as likely pathogenic. Additionally, 10 VUSs and three likely benign imbalances were identified. The results are presented in diagrams in Figure 1. The occurrence of pathogenic and probably pathogenic CNVs was higher in patients with ASD and additional clinical features.

Among the 14 (71.4%) patients with pathogenic and likely pathogenic variants (three females and 11 males), ten were tested by parental diagnostics. Four of these variants were found to be inherited from the mother and five had arisen *de novo*. Importantly, in one case, only the mother was available for diagnostics and maternal origin of the variant was excluded.

Out of the 27 patients with reported variants of uncertain significance, 20 underwent parental diagnostics (74.1%). Of the 20, three patients were found to have *de novo* variants, while nine harbored variants of maternal origin and six of paternal origin. In other cases, the parents did not choose to be tested or were unavailable. The exact list of patients with reported aberrations, variant size, origin if known, and clinical features, are presented in Table 1. Variants classified as benign were not reported in the results. All variants discussed in this manuscript have been submitted to ClinVar, and requests for access to raw sequence data can be made via direct contact with the corresponding author. Records on unique variants identified by our laboratory are available at https://www.ncbi.nlm.nih.gov/clinvar/submitters/507063/ (accessed on 5 March 2024).

A significant point of our study was that it employed a dedicated 4 × 180 k ASD microarray instead of the 8 × 60 CytoSure Constitutional v3 arrays, average resolution 120 kb, which are used in routine testing. Among the reported imbalances, 12 (four likely pathogenic, five VUSs, and three likely benign) would not have been detected using the standard whole-genome microarray. The list of these variants is shown in Table 2.

### 3.1. Pathogenic and Likely CNVs

Importantly, of all the detected pathogenic variants, seven are known to be involved with microduplication or microdeletion syndromes, as is one probably pathogenic variant. Some of the clinical features of these syndromes are autistic traits. Other pathogenic variants identified in this study involve the genes *MET*, *GRIN2A*, and *USP7*. Similarly, the association between ASD and these genes has also been noted in the literature or public databases [34,35]. Among these relevant findings, four involved chromosome 16 and two of them were in the same critical region. Pathogenic and likely pathogenic variants reported in this study are given below:

A 2.61 MB duplication within chromosome 22, which overlaps with 22q11.2 duplication syndrome. The clinical picture of this syndrome is quite variable and some features are shared with 22q11 deletion syndrome; these include heart defects, intellectual disability, dysmorphia, and autism spectrum disorders. Moreover, incomplete penetrance implies that 22q11.2 duplication may occur in asymptomatic individuals and be inherited from healthy parents [36,37].

A 1.36 Mb deletion of 16p13.11. This aberration overlaps intervals I and II of 16p13.11 microdeletion syndrome. The most commonly described features associated with this syndrome are mental retardation, autism, epilepsy, psychiatric disorders, and dysmorphic features.

A 3.44 Mb deletion can occur in the region of 16p13.11 microdeletion syndrome [38,39].

A 202.98 kb deletion can occur at 16p11.2. This deletion, in chromosome 16, is located in the distal part of 16p11.2 microdeletion syndrome with break points BP2-BP3. Patients with this aberration demonstrate psychomotor development delay, autism, obesity, and behavioral disorders. It is associated with incomplete penetrance and variable expression [40].

A 2.77 Mb deletion at 1q21.1q21.2 involving 1q21.1 syndrome. Patients with this syndrome often experience delayed development, microcephaly, ASD, and intellectual disability and display incomplete penetrance and variable phenotype [41].

A 1.48 Mb deletion located in the 17q12 microdeletion syndrome region. Patients with copy number loss within this chromosome fragment have genital tract and kidney abnormalities. If the patient presents with diabetes, it is diagnosed as RCAD. The most common neurological problems are autism spectrum disorders, development and speech delay, intellectual disability, and epilepsy [42].

A 450.97 kb deletion in chromosome 15. This aberration is located in the region of the 15q11.2 microdeletion syndrome, limited by breakpoint BP1-BP2 and encompassing clinically relevant genes like *TUBGC5*, *CYFIP1*, *NIPA1*, and *NIPA2*. In the literature, this kind of deletion is described in patients with ASD, developmental delay in psychomotor and speech, seizures, and ADHD [43].

A 1.11 Mb deletion in 16p13.2. This partially encompasses the *GRIN2A* and *USP7* genes associated with neurodevelopmental disorders, including autistic features [35,44].

A 4.04 Mb deletion in del 7q31.1q31.2. This includes dose-sensitive genes such as *MET*, which is a candidate gene in autism [34]. This deletion has been reported in individuals with autism spectrum disorders, hyperactivity, and short attention span [45].

Six likely pathogenic variants were reported in the ASD patients with comorbidities and one in a patient with isolated autism. All of these variants contain genes associated with ASD to various degrees, viz. *BRWD3*, *NSDHL*, *HSD17B4*, and *PAFAH1B1,* and one pertains to 17p13.3 microduplication syndrome [46,47,48,49]. These variants include a number of genes known to play a role, or a potential one, in the pathogenesis of ASD.

A 212.03 kb deletion of exons 5–7 of the *NRXN1* gene in chromosome 2. Various intragenic heterozygous deletions are described in patients with ASD and intellectual disability and other clinical features such as delayed speech development, ADHD, and epilepsy. No clear data exist regarding this deletion. Exonic deletions in the *NRXN1* gene have different classifications, which may be due to incomplete penetrance. Exon deletions >−5 are considered to have lower penetrance. Nevertheless, the *NRXN1* gene is associated with neurodevelopmental disorders, including autism, and the detected deletion is most likely pathogenic [50].

A 284 bp duplication in Xq21.1. This duplication is located within exon 41 of the *BRWD3* gene. Mutations of this gene are associated with mental retardation, behavioral disturbances, facial dysmorphia and macrocephaly. Duplications have been described in patients with autistic traits [51].

A 50.3 kb Xq28 duplication including the *NSDHL* gene. Mutations in this gene are associated with neurodevelopmental disorders such CHILD syndrome and CK syndrome. Interestingly, a case has been reported of an ASD patient with a duplication of *NSDHL*. Therefore, such changes may well have an influence on patient phenotype [52].

A 190.06 kb deletion in the 5q23.1 region which partially encompasses the *HSD17B4* gene. The *HSD17B4* gene is directly linked to neurological disorders such as Perrault syndrome, which is inherited in an autosomal recessive manner. Previous reports suggest a correlation between alterations in the *HSD17B4* gene and autism spectrum disorders [53].

A 186.62 kb microduplication in the 17p13.3 region, which also seems to have an influence on phenotype. This duplication encompasses the *PAFAH1B* gene whose mutations and deletions are associated with lissencephaly and Miller–Dieker lissencephaly syndrome. Furthermore, duplications have been reported to cause mild structural brain changes and neurodevelopmental disorders, including ASD [48,54].

A 339.09 kb duplication in 2p25.3. This duplication affects exons 3–19 of the *MYT1L* gene, which is associated with neurodevelopmental disorders. Reports in the literature indicate that deletions within *MYT1L* are usually associated with intellectual disability, and duplications with schizophrenia. However, some cases of partial duplication of *MYT1L* were noted in patients with ASD, and hence we regard it as a likely pathogenic [55].

### 3.2. Variants of Uncertain Significance

The variants of uncertain significance identified herein include some noteworthy rare deletions or duplications; however, their classification presents a major challenge in diagnostics. In the present study, based on available databases and literature reports, the identified variants were qualified as a VUS and required further familial diagnostics. Some of these VUSs found in patients with isolated ASD or ASD with additional clinical features are given below:

A 3.63 kb intragenic deletion. This variant is located in Xp21.3 in intron 2 of *IL1RAPL1* gene. Deletions or mutations of this gene have been described in patients with intellectual disability and autistic traits; they are believed to be inherited recessively in association with the X chromosome. Although the described cases include deletions of exons and introns, it remains uncertain whether such changes influence the phenotype [49].

A 2.89 kb rare intragenic deletion in the *PCDH19* gene was described. This deletion is located in intron 3 of the gene. As no similar deletion was found in the available databases nor in the literature, it was classified as a VUS. This gene is located on the X chromosome, and mutations and deletions are associated with epilepsy, developmental and epileptic encephalopathy, and intellectual disability. However, there are some literature reports of patients with autistic traits who present with exonic mutations and deletions of the *PCDH19* gene [56].

A 44.57 kb deletion within the *FOXP1* gene located on chromosome 3. Mutations or deletions in the *FOXP1* gene are associated with the occurrence of a wide range of disorders such as intellectual disability, autism spectrum disorders, hypotonia, dysmorphia, and heart disease. As the deletion was recognized in a noncoding sequence of the gene, it was classified as being of uncertain significance [57].

A 10.24 kb duplication in exon 24 of the *CNTNAP2* gene. *CNTNAP2* has been associated with neurological disorders such as epilepsy, intellectual disability, and autism, especially with language impairment. The literature reports that mutations and deletions within this gene are present in patients with autistic features [58,59].

A 409.66 kb deletion in 11p15.5. This aberration contains many genes, but the most relevant is *DEAF1*, whose mutations are associated with neurodevelopmental disorders and the autosomal dominant Vulto–van Silfhout–de Vries syndrome. Deletions within this gene have been reported in the DGV database, but no cases were noted in the literature [60].

## 4. Discussion

The aim of the study was to identify copy number variants (CNVs) in autism-related genes and in candidate genes that may be significant in the etiology of autism. Samples from a group of patients presenting isolated ASD, or ASD with additional clinical features, were studied by molecular karyotyping.

Our overall diagnostic yields across the entire group were 7.8% for clinically significant variants and 8.9% for those of uncertain clinical significance; these values are close to those obtained by Lovrecić et al., i.e., 7.3% and 10%, respectively. However, in contrast to our study, Lovrecić et al. examined 150 patients, divided into three groups: the first comprising ASD patients with or without additional behavioral characteristics, the second including ASD patients with a developmental delay, and the third with ASD and other complex clinical features, such as congenital anomalies, epilepsy, or intellectual disability. The study also employed CGH ISCA v2 8 × 60 K microarrays with a practical resolution of 100 kb [4].

Annunziata et al. examined the prevalence of CNVs in a group of 209 autistic children using oligo ISCA180 K microarrays. The patients were divided into two groups: either isolated ASD or complex ASD with more than six facial and somatic characteristics. Despite achieving a lower diagnostic rate for pathogenic variants, i.e., 5.2% in the whole group, they did not identify any likely pathogenic variants, which were classified as significant in the present study. However, Annunziata et al. report a 12.8% prevalence of clinically relevant variants among patients presenting with complex autism compared to 10.5% in the present study; they also note a 3.1% diagnostic rate of relevant variants in patients with isolated autism compared to 4% in our present study [15].

Miles et al. define essential ASD as the presence of autism lacking any clinical features associated with an abnormal morphogenesis, and complex ASD as autism accompanied by numerous physical anomalies and/or microcephaly [12]. Based on this distinction, a study by Napoli et al. examined the presence of CNVs in a group of 133 patients with ASD using Agilent Human Genome 4 × 180 k CGH microarrays with a resolution of around 75 kb. The detected CNVs were divided into C-CNVs (causative copy number variations), NC-CNVs (non-causative copy number variations), and W-CNVs (without copy number variations). The prevalence of pathogenic and potentially pathogenic CNVs was 9.02% among patients with isolated ASD, i.e., higher than the respective values noted in the present study, by Lovrečić et al., and by Annunziata et al. [61]. It is important to note that although each of the cited studies, and the present one, used different microarrays, they all identified similar frequencies of pathogenic variants and VUSs. Indeed, these studies report similar prevalences of pathogenic, probably pathogenic variants and VUSs as our present findings, and the changes often involved the same genes. However, the classification may differ depending on whether a gain or loss occurs, or which chromosome or gene fragment is involved.

The rate of detection is also influenced by the type of diagnostic tool. The present study used a 4 × 180 k CytoSure Autism Research Array dedicated to autism spectrum disorders. Interestingly, a closer review of our data indicated 12 out of the 40 identified pathogenic variants and VUSs (30%) would not have been found using an 8 × 60 k microarray; furthermore, these 12 variants included four likely pathogenic, five VUSs, and three likely benign variants. This finding underscores the importance of proper clinical evaluation and accurate selection of a suitable diagnostic tool for CNV analysis. The interpretation of copy number variations, particularly those of unknown clinical significance, remains a major challenge in diagnostics [23]. The correct identification of CNVs depends on many factors, and classification can be complicated by a lack of information about the patient’s phenotype: such data is needed to relate role of the protein encoded by the gene to the phenotype. In such cases, the clinician has a particularly important role. This understanding can also be influenced by the prevalence of the variant in the population and dosage sensitivity. Most importantly, when using public databases, it is important to bear in mind that the size of the CNV may also be influenced by the use of particular types of microarray, i.e., with different probe densities and arrangements. Furthermore, many databases do not provide the sex of the patient, which makes it difficult to analyze variants on the X chromosome.

Furthermore, in clinical practice, when determining the segregation between phenotypes in a family, an important role is also played by the diagnostic results of the parents. However, even if a variant was inherited from a parent who does not have the same phenotype as their child, this does not provide certainty about the significance of the variant; even related individuals can demonstrate incomplete penetration and variable expression of phenotypic traits. Such differences can also be caused by the presence of a variant on the second allele of a particular gene, even in the region of the CNV, which can result in disease symptoms. As such, it is important to take particular care when reporting copy number variations of unknown clinical significance [62].

Thanks to the rapid development of genetic diagnostics, a number of candidate genes whose variants increase susceptibility to ASD have been identified; many studies also indicate that copy number variation appears to be a risk factor in ASD. As such, it is impossible to confirm whether the reported variants with unknown pathogenicity have any clinical impact. Such cases require a precise genotype–phenotype analysis of the family of the proband, together with a comprehensive phenotypic evaluation. It is also important to note that with the ongoing growth of knowledge in this area and acquisition of data, the classification of the variants continues to develop; as such, the acquired genomic data should be subjected to further re-evaluation of using high-resolution, genomic analyses with the aim of clarifying the molecular basis of ASD. Our present findings, based on patients with a diagnosis of ASD with or without additional clinical features, demonstrate that aCGH has high clinical value compared to classical karyotyping [61,62].

Importantly, the rapid development of sequencing techniques has demonstrated the potential for whole exome sequencing (WES) in diagnostics. Although sequencing was originally dedicated to the detection of single nucleotide variants (SNVs), advances in bioinformatics have allowed the use of tools for copy number analysis based on sequencing data. There are now a range of tools with various degrees of sensitivity, accuracy, and performance that can detect CNVs based on WES data. Such procedures allow simultaneous analysis of both single variants and CNVs based on data from a single procedure. Furthermore, such analysis significantly reduces the cost of the test, particularly considering the expense of dual diagnostics, such as SNV assessment by custom panel sequencing and additionally CNV identification by microarray. The single procedure also allows improved diagnostic performance and shorter time to diagnosis. However, despite the rapid development of the WES technique and bioinformatics tools, microarrays are still considered the gold standard in copy number testing, and at the time of the study, WES was not regarded as a routine technique for detecting CNV in our laboratory. Therefore, the microarray technique was an appropriate choice in the present study [63,64].

## 5. Conclusions

Our results indicate that the standard microarrays in routine use may not have sufficient resolution to reliably assess patients with ASD. Therefore, future research should consider the use of more targeted diagnostic tools, such as dedicated high-resolution microarrays. However, the first priority is to perform a comprehensive assessment of the genotype-phenotype correlation by the combination of whole exome sequencing with CNV analysis. Although it may be fundamental to predict phenotypes from genotypes in biology, it is very challenging in practice. As some variants of specific genetic diseases may be associated with incomplete penetrance or variable expressivity, especially in relation to VUS, it may not be possible to establish a definitive genotype–phenotype correlation; in some cases, an alteration may confer only a susceptibility to a certain disorder. As such, it is important to report VUSs in publications. The number of VUSs is likely to decrease, as their significance is being increasingly recognized in the medical literature. Therefore, we hope that our results will be of value in improving the general understanding of the genotype-phenotype relationship of ASD.

## 6. Limitations

Patients were recruited on the basis of diagnosis according to ICD-10; however, neither the ICD-10 nor the newer ICD-11 take into account the division into mild and severe ASD.

In addition, the severity of the phenotype was not defined during the recruitment process, and the cases were only classified as isolated autism and autism with additional abnormalities. We are aware that this lack of information regarding ASD severity is a limitation of this manuscript, and one that will be addressed in future studies. For a clearer phenotypical picture and the relevance of CNVs to clinicians, it can be hypothesized that individuals with severe ASD (DSM-5), i.e., autism (DSM-IV), would have more CNV hits, or more pathogenic hits, or both, than patients with mild ASD (PDD NOS in DSM-IV).

The study did not include a typical control group of healthy ethnically and age-matched individuals. Instead, the Database of Genomic Variants (DGV) and other public databases providing a comprehensive summary of structural variation in the human genome were used.

All variants were successfully submitted; however, at this stage temporal submission numbers, but not accession numbers are available.

## Figures and Tables

**Table 1:** A detailed list of pathogenic and likely pathogenic variants, including aCGH result, size of CNV, inheritance (if known), sex, and clinical phenotype.

	Deletion/Duplication	aCGH Result	Size	Classification	Inheritance	Sex	Clinical Features
1.	Duplication	arr[GRCh37] 22q11.21(18842715_21449931)x3	2.61 Mb	Pathogenic	maternal	Female	Autism, speech development delay, dysmorphia
2.	Deletion	arr[GRCh37] 16p13.11(15493513_16288126)x1	1.36 Mb	Pathogenic	unknown	Female	Autism, moderate intellectual disability, dysmorphia, motor and speech development delay
3.	Deletion	arr[GRCh37] 17q12(34821955_36305651)x1	1.48 Mb	Pathogenic	maternal	Male	Autism spectrum disorders, speech development delay, hyperactivity, stereotypic movements
4.	Deletion	arr[GRCh37] 15q11.2(22788634_23239599)x1	450.97 kb	Pathogenic	not maternal	Male	Autism spectrum disorders, severe intellectual disability, epilepsy, stereotypic movements, motor and speech development delay
5.	Deletion	arr[GRCh37] 16p13.11p12.3(15278668_18719513)x1	3.44 Mb	Pathogenic	unknown	Male	Autism, moderate intellectual disability, facial dysmorphia, motor development delay
6.	Deletion	arr[GRCh37] 7q31.1q31.2(113244833_117283945)x1	4.04 Mb	Pathogenic	unknown	Male	Autism, moderate intellectual disability, motor and speech development delay, hyperactivity, problems with concentration
7.	Deletion	arr[GRCh37] 16p13.2(9031580_10143057)x1	1.11 Mb	Pathogenic	unknown	Male	Atypical autism, motor and speech development delay
8.	Deletion	arr[GRCh37] 1q21.1q21.2(145421717_148193211)x1	2.77 Mb	Pathogenic	unknown	Male	Atypical autism, moderate intellectual disability, motor development delay, aggressive behavior
	Deletion	arr[GRCh37] 16p11.2(28836637_29039612)x1	202.98 kb	Pathogenic	unknown		
9.	Deletion	arr[GRCh37] 2p16.3(50817145_51029172)x1	212.03 kb	Likely pathogenic	de novo	Male	Autism spectrum disorders, moderate intellectual disability, motor and speech development delay, overweight
10.	Deletion	arr[GRCh37] 5q23.1(118891108_119081165)x1	190.06 kb	Likely pathogenic	maternal	Male	Autism, epilepsy, speech development delay, hyperactivity
11.	Duplication	arr[GRCh37] Xq21.1(79926786_79927069)x2	284 b	Likely pathogenic	de novo	Male	Autism, facial dysmorphia, paroxysmal EEG, motor and speech development delay
12.	Duplication	arr[GRCh37] Xq28(151987969_152038267)x2	50.3 kb	Likely pathogenic	maternal	Male	Atypical autism, moderate intellectual disability, epilepsy, speech development delay, aggressive behavior, hyperactivity
13.	Duplication	arr[GRCh37] 2p25.3(1856074_2195166)x3	339.09 kb	Likely pathogenic	de novo	Female	Autism, motor and speech development delay
14.	Duplication	arr[GRCh37] 17p13.3(2403985_2590608)x3	186.62 kb	Likely pathogenic	de novo	Male	Atypical autism, epilepsy, motor and speech development delay, hyperactivity, stereotypic movements
	Duplication	arr[GRCh37] 12q24.31(123625555_124063172)x3	437.62 kb	Uncertain significance	de novo		
15.	Deletion	arr[GRCh37] 3q26.31q26.32(175090409_175729002)x1	638.59 kb	Uncertain significance	unknown	Male	Autism, motor and speech development delay, dyspraxia, stereotypic movements
16.	Duplication	arr[GRCh37] 2q12.3q13(109301667_110460985)x3	1.16 Mb	Uncertain significance	paternal	Female	Autism
	Duplication	arr[GRCh37] 5q13.2(70674236_71625281)x3	951 kb	Uncertain significance	de novo		
17.	Duplication	arr[GRCh37] 6q15(89347838_90436358)x3	1.09 Mb	Uncertain significance	paternal	Male	Autism, motor and speech development delay, self-injurious behavior
	Duplication	arr[GRCh37] 15q13.3q14(32932862_34817263)x3	1.88 Mb	Uncertain significance	paternal		
18.	Duplication	arr[GRCh37] 7p15.2(26028362_26535193)x3	506.83 kb	Uncertain significance	paternal	Female	Autism spectrum disorders, motor development delay, lack of speech development, stereotypic behavior
19.	Deletion	arr[GRCh37] Xp21.3(28870305_28873932)x0	3.63 kb	Uncertain significance	unknown	Male	Autism spectrum disorders, speech development delay, hyperactivity
20.	Deletion	arr[GRCh37] Xq22.1(99621729_99624614)x1	2.89 kb	Uncertain significance	paternal	Female	Autism spectrum disorders, regression of speech development, dyspraxia, hyperactivity
21.	Deletion	arr[GRCh37] 14q21.2q21.3(46872015_47631965)x1	760 kb	Uncertain significance	maternal	Male	Autism spectrum disorders, motor and speech development delay, dyspraxia, problems with concentration
22.	Duplication	arr[GRCh37] 5p13.2(33977521_34745571)x3	768 kb	Uncertain significance	maternal	Male	Atypical autism, agenesis of the corpus callosum, motor and speech development delay, stereotypic behavior
	Deletion	arr[GRCh37] 11p15.5(313988_723647)x1	409.66 kb	Uncertain significance	de novo		
23.	Duplication	arr[GRCh37] 18p11.21(12244577_12507814)x3	263 kb	Uncertain significance	maternal	Male	Autism, speech development delay
24.	Duplication	arr[GRCh37] 4q21.21(79643334_80443461)x3	800.13 kb	Uncertain significance	maternal	Female	Autism, motor and speech development delay, hyperactivity, stereotypic and aggressive behavior, macrosomia
25.	Deletion	arr[GRCh37] 2p16.3(50801244_50807336)x1	6.09 kb	Uncertain significance	maternal	Male	Autism spectrum disorders, hypertrophy, hypotonia, craniofacial dysmorphia
26.	Duplication	arr[GRCh37] 7q36.1(148108214_148118453)x3	10.24 kb	Uncertain significance	unknown	Male	Autism, intellectual disability, motor and speech development delay
27.	Duplication	arr[GRCh37] 7q34(140600860_140711281)x3	110.42 kb	Uncertain significance	maternal	Male	Autism, dysmorphia
28.	Deletion	arr[GRCh37] 1q31.2q31.3(193702611_196175057)x1	2.47 Mb	Uncertain significance	paternal	Female	Autism, mild intellectual disability, speech development delay
29.	Duplication	arr[GRCh37] Xq24(118279988_118843305)x2	563.32 kb	Uncertain significance	mat	Male	Autism, speech development delay, hyperactivity
30.	Deletion	arr[GRCh37] 3p13(71567496_71612062)x1	44.57 kb	Uncertain significance	maternal	Male	Autism
31.	Duplication	arr[GRCh37] 10p11.21(35180210_35500482)x3	320.27 kb	Likely benign	unknown	Male	Autism, macrosomia
32.	Duplication	arr[GRCh37] 10p11.21(35180210_35500482)x3	320.27 kb	Likely benign	unknown	Male	Autism, macrosomia
33.	Duplication	arr[GRCh37] 4q35.2(188121998-189791846)x3	1.67 Mb	Likely benign	paternal	Male	Autism, moderate intellectual disability, motor and speech development delay
34.	Deletion	arr[GRCh37] 7p21.3(12915284_13271423)x1	356 kb	Likely benign	unknown	Female	Atypical autism, dysmorphia
35.	Deletion	arr[GRCh37] 7q35(146017736_146036542)x1	18.81 kb	Likely benign	unknown	Female	Autism, epilepsy, dysmorphia

**Table 2:** A list of reported variants, and whether they are also detectable on an 8 × 60 k microarray.

Classification	Deletion/Duplication	Chromosome and Cytoband	Detectability by 8 × 60 CytoSure Constitutional v3 Array: Yes/No
pathogenic	duplication	22q11.21	yes
pathogenic	deletion	16p13.11	yes
pathogenic	deletion	17q12	yes
pathogenic	deletion	15q11.2	yes
pathogenic	deletion	16p13.11p12.3	yes
pathogenic	deletion	7q31.1q31.2	yes
pathogenic	deletion	16p13.2	yes
pathogenic	deletion	1q21.1q21.2	yes
pathogenic	deletion	16p11.2	yes
likely pathogenic	deletion	2p16.3	no
likely pathogenic	deletion	5q23.1	no
likely pathogenic	duplication	Xq21.1	no
likely pathogenic	duplication	Xq28	yes
likely pathogenic	duplication	17p13.3	yes
likely pathogenic	duplication	2p25.3	no
VUS	deletion	3q26.31q26.32	no
VUS	duplication	2q12.3q13	yes
VUS	duplication	5q13.2	yes
VUS	duplication	6q15	yes
VUS	duplication	15q13.3q14	yes
VUS	duplication	7p15.2	yes
VUS	deletion	Xp21.3	no
VUS	deletion	Xq22.1	yes
VUS	deletion	14q21.2q21.3	yes
VUS	duplication	5p13.2	yes
VUS	deletion	11p15.5	yes
VUS	deletion	2p16.3	no
VUS	duplication	7q36.1	no
VUS	duplication	12q24.31	yes
VUS	duplication	7q34	no
VUS	duplication	Xq24	yes
VUS	duplication	18p11.21	yes
VUS	duplication	4q21.21	yes
VUS	deletion	1q31.2q31.3	yes
VUS	deletion	3p13	yes
likely benign	duplication	10p11.21	no
likely benign	duplication	10p11.21	no
likely benign	duplication	4q35.2	yes
likely benign	deletion	7p21.3	yes
likely benign	deletion	7q35	no

## Data Availability

All data presented in this study are available on reasonable request from the corresponding author. The data are not publicly available due to the presence of the private information of individuals.
