# Correction: Kucińska et al. The Use of CGH Arrays for Identifying Copy Number Variations in Children with Autism Spectrum Disorder. *Brain Sci.* 2024, *14*, 273

**DOI:** 10.3390/brainsci14060529

**Published:** 2024-05-22

**Authors:** Agata Kucińska, Wanda Hawuła, Lena Rutkowska, Urszula Wysocka, Łukasz Kępczyński, Małgorzata Piotrowicz, Tatiana Chilarska, Nina Wieczorek-Cichecka, Katarzyna Połatyńska, Łukasz Przysło, Agnieszka Gach

**Affiliations:** 1 Department of Genetics, Polish Mother’s Memorial Hospital-Research Institute, 93-338 Lodz, Poland; wanda.hawula@iczmp.edu.pl (W.H.); lena.rutkowska@iczmp.edu.pl (L.R.); urszula.wysocka@iczmp.edu.pl (U.W.); lukasz.kepczynski@iczmp.edu.pl (Ł.K.); malgorzata.piotrowicz@iczmp.edu.pl (M.P.); tatiana.chilarska@iczmp.edu.pl (T.C.); nina.wieczorek-cichecka@iczmp.edu.pl (N.W.-C.); agnieszka.gach@iczmp.edu.pl (A.G.); 2 Department of Developmental Neurology and Epileptology, Polish Mother’s Memorial Hospital-Research Institute, 93-338 Lodz, Poland; katarzyna.polatynska@iczmp.edu.pl (K.P.); lukasz.przyslo@iczmp.edu.pl (Ł.P.)

## Missing Citation

In the original publication [1], in the last paragraph of the discussion, there should also be an insertion of a new reference citation [64] after [63]. The final paragraph of the Discussion and Pre-Processing Sections and should read in the following way:

Therefore, the microarray technique was an appropriate choice in the present study [63,64].

## Text Correction

All the changes to the bibliography have meant that in the main text of the manuscript, the references starting from number [27] should be changed to be one number higher, starting from [27] to [28], then [28] to [29] and so on until the end of the list.

In the original publication [1], the fourth paragraph of the introduction citation following the first sentence is numbered [21] and should be renumbered to [27] Hnoonual, A.; Thammachote, W.; Tim-Aroon, T.; Rojnueangnit, K.; Hansakunachai, T.; Sombuntham, T.; Roongpraiwan, R.; Worachotekamjorn, J.; Chuthapisith, J.; Fucharoen, S.; et al. Chromosomal Microarray Analysis in a Cohort of Underrepresented Population Identifies SERINC2 as a Novel Candidate Gene for Autism Spectrum Disorder. *Sci. Rep.* **2017**, *7*, 12096.

In the last paragraph of the results, reference [12] should be replaced with [60] Nabais Sá, M.J.; Jensik, P.J.; Mcgee, S.R.; Parker, M.J.; Lahiri, N.; Mcneil, E.P.; Kroes, H.Y.; Hagerman, R.J.; Harrison, R.E.; Montgomery, T.; et al. De Novo and Biallelic DEAF1 Variants Cause a Phenotypic Spectrum. *Genet. Med.* **2019**, *21*, 2059–2069.

In the fourth paragraph of the discussion, following the first sentence, the reference [59] should be replaced with [12] Miles, J.H.; Takahashi, T.N.; Bagby, S.; Sahota, P.K.; Vaslow, D.F.; Wang, C.H.; Hillman, R.E.; Farmer, J.E. Essential versus Complex Autism: Definition of Fundamental Prognostic Subtypes. *Am. J. Med. Genet.* **2005**, *135A*, 171–180, doi:10.1002/ajmg.a.30590.

## References List Correction

In the reference list section, add two new references [20] Lozano, R.; Rosero, C.A.; Hagerman, R.J. Fragile X Spectrum Disorders. *Intractable Rare Dis. Res.* 2014, 3, 134–146; and [21] Lozano, R.; Hagerman, R.J.; Duyzend, M.; Budimirovic, D.B.; Eichler, E.E.; Tassone, F. Genomic studies in fragile X premutation carriers. *J. Neurodev. Disord.* **2014**, *6*, 27; after reference [19].

Insert original references [20] Zarrei, M.; MacDonald, J.R.; Merico, D.; Scherer, S.W. A Copy Number Variation Map of the Human Genome. *Nat. Rev. Genet.* **2015**, *16*, 172–183. and [21] Haraksingh, R.R.; Abyzov, A.; Urban, A.E. Comprehensive Performance Comparison of High-Resolution Array Platforms for Genome-Wide Copy Number Variation (CNV) Analysis in Humans. *BMC Genom.* **2017**, *18*, 321. after reference [22].

As the reference numbers used start from [23] and increase by two, the final corrected reference number range has been amended to [25–64].

The authors state that the scientific conclusions are unaffected. This correction was approved by the Academic Editor. The original publication has also been updated.
